# Artificial Neural Network-Based Ultrasound Radiomics Can Predict Large-Volume Lymph Node Metastasis in Clinical N0 Papillary Thyroid Carcinoma Patients

**DOI:** 10.1155/2022/7133972

**Published:** 2022-06-17

**Authors:** Wan Zhu, Xingzhi Huang, Qi Qi, Zhenghua Wu, Xiang Min, Aiyun Zhou, Pan Xu

**Affiliations:** ^1^Departments of Ultrasonography, The First Affiliated Hospital of Nanchang University, Nanchang, Jiangxi, China; ^2^Departments of Head and Neck Otolaryngology, The First Affiliated Hospital of Nanchang University, Nanchang, Jiangxi, China

## Abstract

**Objective:**

To evaluate the ability of artificial neural network- (ANN-) based ultrasound radiomics to predict large-volume lymph node metastasis (LNM) preoperatively in clinical N0 disease (cN0) papillary thyroid carcinoma (PTC) patients.

**Methods:**

From January 2020 to April 2021, 306 cN0 PTC patients admitted to our hospital were retrospectively reviewed and divided into a training (*n* = 183) cohort and a validation cohort (*n* = 123) in a 6 : 4 ratio. Radiomic features quantitatively extracted from ultrasound images were pruned to train one ANN-based radiomic model and three conventional machine learning-based classifiers in the training cohort. Furthermore, an integrated model using ANN was constructed for better prediction. Meanwhile, the prediction of the two models was evaluated in the papillary thyroid microcarcinoma (PTMC) and conventional papillary thyroid cancer (CPTC) subgroups.

**Results:**

The radiomic model showed better discrimination than other classifiers for large-volume LNM in the validation cohort, with an area under the receiver operating characteristic curve (AUROC) of 0.856 and an area under the precision-recall curve (AUPR) of 0.381. The performance of the integrated model was better, with an AUROC of 0.910 and an AUPR of 0.463. According to the calibration curve and decision curve analysis, the radiomic and integrated models had good calibration and clinical usefulness. Moreover, the models had good predictive performance in the PTMC and CPTC subgroups.

**Conclusion:**

ANN-based ultrasound radiomics could be a potential tool to predict large-volume LNM preoperatively in cN0 PTC patients.

## 1. Introduction

Papillary thyroid carcinoma (PTC) is the most common pathological type of thyroid cancer. According to the World Health Organization (WHO), PTC with a maximum diameter of 10 mm is defined as papillary thyroid microcarcinoma (PTMC), and those with a maximum diameter of more than 10 mm are called conventional papillary thyroid cancer (CPTC) [1, 2]. Even though PTC is considered an indolent tumor, approximately 30%–80% of PTC patients would present with central lymph node metastasis (LNM). Clinically LN-positive (clinical N1 disease, cN1) cases are becoming more frequent due to increased ultrasound examination and more meticulous examination of surgical specimens by pathologists [3]. Nonetheless, 30%–65% of clinically *N*-negative (clinical N0 disease, cN0) PTC patients are detected with LNM postoperatively [4].

However, not all PTCs with LNM have a poor prognosis. The recurrence rate of patients with small-volume LNM (≤5 involved LNs) (median 4%, range 3%–8%) is significantly lower than that of patients with large-volume LNM (>5 involved LNs) (median 19%, range 7%–21%) [5, 6]. In addition, upstaging risk of the PTC based on the detection of microscopic locoregional metastases may result in more aggressive treatment [7, 8]. Even a single-microscopic LN metastasis can upstage a patient with low-risk PTC to intermediate risk of recurrence in the American Thyroid Association (ATA) system and an increased risk of death in the American Joint Committee on Cancer (AJCC) staging system [7, 8]. Therefore, an accurate preoperative predictive tool for large-volume LNM can more precisely guide treatment.

Previous studies reported that age (<40 years old) and male sex were significantly associated with large-volume LNM in PTC patients [9–11]. Nevertheless, these studies focused on identifying risk factors for large-volume LNM rather than constructing a predictive model. Ultrasound is the first-line noninvasive imaging method for cervical LNM, with a specificity of 85.0%–97.4% but a sensitivity of 20%–31% [12, 13]. Radiomics can identify high-throughput quantitative imaging features and discover information reflecting the underlying pathophysiology that cannot be assessed by visual interpretation [14]. In recent years, radiomics based on ultrasound has been deemed to have a good predictive ability for cervical LNM in PTC patients [15].

An artificial neural network (ANN) is a complex network with many simple components connected, which can perform complex logical operations and identify nonlinear relationships; thus, it has been applied in machine learning-based radiomics model construction [16]. This study developed and validated two predictive models that could adequately combine the ANN and ultrasound radiomics to predict large-volume LNM in cN0 PTC patients.

## 2. Materials and Methods

The review board of the First Affiliated Hospital of Nanchang University approved this retrospective study. A retrospective review with deidentified data was used, and no protected health information was acquired. Thus, the need for informed consent from all patients was waived.

### 2.1. Patients

From January 2020 to April 2021, patients with PTC admitted to the Department of Otolaryngology in our hospital were enrolled. The inclusion criteria were as follows: (1) patients treated through total thyroidectomy with bilateral central lymph node dissection (CND), with pathological results being available; (2) ultrasound examination performed within two weeks before surgery; (3) availability of ultrasound images of the target nodule in the most extended axis cross section; (4) more than 18 years old. The exclusion criteria were as follows: (1) no more than five lymph nodes (LNs) resected; (2) met cN1 diagnostic criteria preoperatively; (3) target nodule treated through radiofrequency ablation, radiotherapy, or chemotherapy before ultrasound examination; (4) target nodule unclear on ultrasound images due to artifacts; (5) accompanied by other diseases that can lead to pathological N-positive. In this study, cN1 was defined by at least one of the following features obtained during preoperative ultrasound examination: the ratio of transverse/long diameter >0.5, blurred corticomedullary boundary, vanished medulla structure, microcalcification, cystic changes, and chaotic or peripheral vascular pattern microcalcification [17–19].

**Figure 1:**
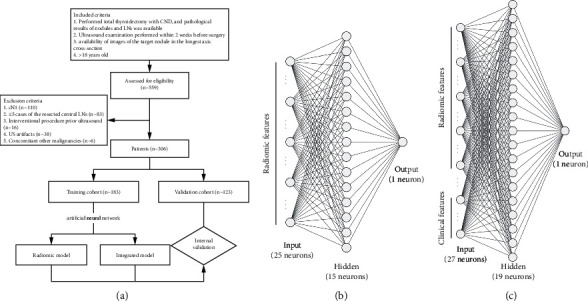
Flowcharts of the patient selection (a), schematic representation of the ANN-based radiomic model (b), and ANN-based integrated model (c). ANN, artificial neural network; CND, central lymph node dissection; LN, lymph node.

A total of 559 patients met the inclusion criteria, and 306 patients (median age 45 years, range 24–81 years; 65 men and 241 women) were enrolled after exclusion (Figure 1(a)). Among these patients, 156 patients were reported in our previous studies, which developed and validated an ultrasound radiomic model for predicting malignant thyroid nodules [20]. All patients were randomly divided into the training cohort (*n* = 183) and validation cohort (*n* = 123) in a 6 : 4 ratio.

### 2.2. Clinical and Ultrasound Information

Baseline clinicopathological data, including age, sex, and pathology of the nodule and LN, were obtained from medical records. Patients were divided into two groups by age (age <40 years and ≥40 years old) [10, 11]. Ultrasound Digital Imaging and Communications in Medicine (DICOM) images were acquired with a Philips iU Elite and EPIQ7 (ultrasound system, Philips Medical System, Bothell, WA, USA) using a 5–12 MHz linear transducer. Two radiologists with over 5 years and 8 years of experience were blinded to the pathological results and reviewed the images using Picture Archiving and Communication Systems (PACS). They evaluated the 2017 American College Radiology (ACR) Thyroid Imaging Reporting and Data System (TI-RADS) [21], tumor size, and capsule invasion in consensus. The nodule with the highest ACR score was selected as the target nodule in the case of multifocality; when the scores of nodules were the same, the larger diameter nodule was selected.

### 2.3. Nodule Segmentation and Feature Extraction

Two radiologists with over 3 years and 10 years of experience, blinded to the pathological results and corresponding LN images, manually segmented the region of interest (ROI) of the target nodule using 3D Slicer version 4.10.2 open-source software (3D Slicer, version 4.10.2; National Institutes of Health-funded; https://www.slicer.org) (Supplementary Materials 1) [22]. A single representative section with the largest nodule area was chosen for the nodular ROI. The intraobserver and interobserver agreements were evaluated using 30 randomly chosen nodules delineated by a radiologist twice within two weeks and by another radiologist. The mean intraclass correlation coefficient (ICC) > 0.75 represented satisfactory agreement. A radiologist delineated the remaining nodules if a strong agreement (mean ICC >0.90) was achieved. Open-source software (PyRadiomics 3.0.1; http://pyradiomics.readthedocs.io/en/latest/index.html) [23] extracted 849 radiomic texture, shape, and intensity features from the original and wavelet-filtered images of each nodule (Supplementary Materials 2). Resampling and *z* score normalization were performed as preprocessing steps.

### 2.4. Radiomic Feature Dimension Reduction and Selection

To resolve the data imbalance, we used SMOTE to balance the training cohort [24]. Dimensionality reduction and radiomic feature selection were performed in the following steps: (1) radiomic features with intraobserver or interobserver ICC no more than 0.75 were removed; (2) radiomic features were excluded due to insignificant differences based on univariate analysis (Mann–Whitney *U* test); (3) Spearman's correlation coefficient (*r*) was used to assess the correlations among all radiomic features, and highly correlated features (>0.80) and those with a lower area under the receiver operator characteristic curve (AUROC) were removed; (4) we applied the least absolute shrinkage and selection operator (LASSO) method [25, 26] to select the most significant features.

### 2.5. Radiomic Model and Integrated Model Construction

Based on significant radiomic features, we built a single-layer, feed-forward ANN with a backpropagation algorithm to build a radiomic model for large-volume LNM using the data of the training cohort. These radiomic features were used to train linear discriminant analysis, support vector machine, and random forest classifiers.

To provide a more practical tool for prediction, we assessed the incremental value of clinical data as an additional predictor. Clinical factors with *p* < 0.05 according to univariate and multivariate logistic regression analyses were considered independent risk factors. We established a clinical model using multivariate logistic regression for comparison. Then, an ANN integrated model incorporating radiomic features and independent clinical risk factors was constructed. Supplementary Materials 3 shows detailed ANN training. The probabilities predicted by the radiomic model and integrated model were called Rad-prob and Inte-prob, respectively. ROC may portray an overly optimistic performance on account of our data imbalance; thus, we applied the precision-recall (PR) curve simultaneously, which can focus on the minority class [27]. According to the PR curve, the optimal cut-off value was defined as the probability that yields the max sum of precision and recall in the training cohort.

### 2.6. Radiomic Model and Integrated Model Validation

AUROC and area under the PR curve (AUPR) evaluated the ANN-based and three conventional machine learning-based classifiers on the validation cohort. Predictive performance was assessed for radiomic and integrated models, including discrimination, calibration, and clinical usefulness. The Hosmer–Lemeshow test and calibration curve were evaluated for calibration [28]. The discrimination metrics included accuracy, sensitivity, specificity, positive predictive value (PPV), negative predictive value (NPV), AUROC, and AUPR. Decision curve analysis (DCA) was conducted to determine the clinical usefulness by quantifying the net benefits at different threshold probabilities.

### 2.7. Model Validation in PTMC and CPTC

The entire cohort was divided into the PTMC subgroup (≤10 mm; *n* = 114) and CPTC subgroup (>10 mm; *n* = 192) by the maximum diameter. Through subgroup analysis, we investigated whether patients with large-volume LNM could be predicted in the subgroups using the radiomic model and integrated model. The performance metrics included accuracy, sensitivity, specificity, PPV, NPV, AUROC, and AUPR.

### 2.8. Statistical Analysis

Statistical analyses were performed using Python (Version 3.8.8; https://www.python.org/) and *R* (Version 4.0.1, https://www.r-project.org/). Continuous variables were expressed as medians with interquartile ranges (IQRs) and compared using the Mann–Whitney *U* test, and categorical data were expressed as numbers with percentages and compared using the chi-square test or Fisher's exact test. The Delong test was used to compare the AUROCs. All statistical tests were two-sided, and *p* < 0.05 was considered statistically significant.

## 3. Results

### 3.1. Patient Clinicopathological Characteristics

The baseline clinicopathological characteristics are presented in Table 1. The analysis showed no significant differences in clinicopathological characteristics between the training and validation cohorts. PTCs with large-volume LNM accounted for 10.4% (19/183) and 8.9% (11/123) of the training and validation cohorts, respectively (*p*=0.827). The characteristics of the patients according to their large-volume LNM status are listed in Table 2. Younger age (<40 years) and male sex were significantly associated with a higher prevalence of large-volume LNM (all *p* < 0.05).

### 3.2. Radiomic Feature Dimension Reduction and Selection

**Figure 2:**
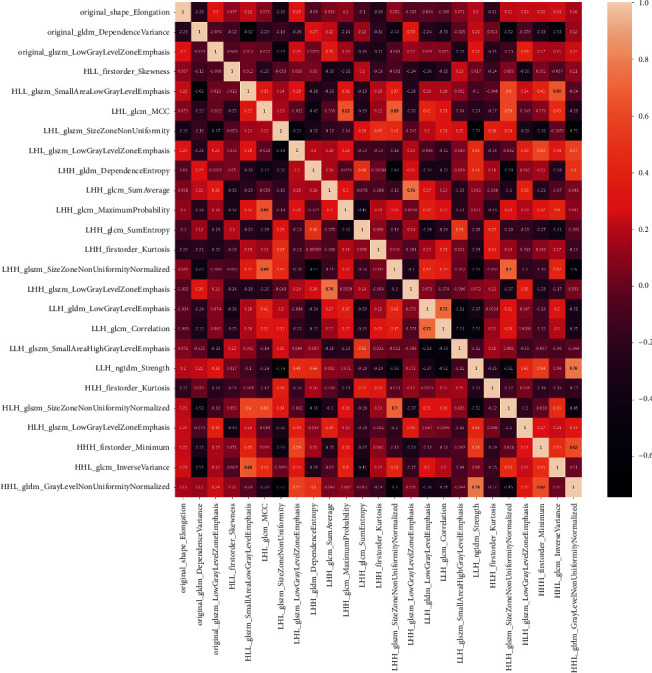
Heatmap of pairwise Spearman correlations between the selected radiomic features after feature reduction in the training cohort.

The rates of intraobserver and interobserver agreement for the radiomics features reached 95.1% (807/849; mean ICC = 0.950) and 95.6% (812/849; mean ICC = 0.941), respectively (Supplementary Materials 4). Forty-four radiomic features were excluded due to unsatisfactory agreement, and 145 were excluded due to insignificant differences based on univariate analysis. After the correlation analysis, 58 features remained. Then, 25 radiomic features were selected as the most significant features for predicting large-volume LNM by LASSO regression (Supplementary Materials 5). The names of the features and heatmap of the pairwise Spearman correlations are shown in Figure 2.

### 3.3. Radiomic Model and Integrated Model Construction

Our ANN-based radiomic model consisted of 25 input radiomic feature variables, 15 neurons in the 1st hidden layer, and 1 output unit that can obtain each probability of large-volume LNM (Figure 1(b). Rad-prob had an accuracy of 86%, an AUROC of 0.890, and an AUPR of 0.348 in the training cohort.

In multivariate logistic regression, age (<40 years) (odds ratio (OR) 3.59, 95% confidence interval (CI) 1.31–9.87; *p*=0.013) and male sex (OR 3.72, 95% CI 1.36–10.18; *p*=0.011) were independent risk factors for large-volume LNM. With additional 2 input clinical factors, the integrated model was constructed (Figure 1(c); Supplementary Materials 6 for the training and testing loss and accuracy curves). The accuracy, AUROC, and AUPR of Inte-prob significantly increased to 91%, 0.910, and 0.463 (Table 3).

### 3.4. Radiomic Model and Integrated Model Validation

**Figure 3:**
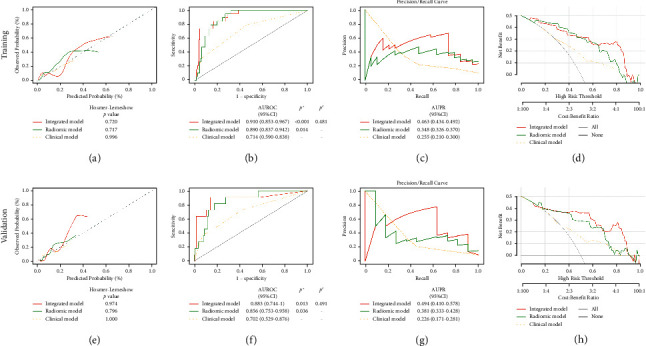
Predictive performance of the radiomic model and integrated model. Calibration curves (a, e), ROC curves (b, f), PR curves (c, g), and DCA (d, h) of the models. ^*∗*^Statistically different (Delong test) from the clinical model. ^#^Statistically different (Delong test) from the radiomic model. AUPR, area under the precision-recall curve; AUROC, area under the receiver operator characteristic curve; CI, confidence interval; DCA, decision curve analysis.

In the validation cohort, the AUROC and AUPR of the ANN-based radiomic model were higher than those of three conventional machine learning-based classifiers (detailed ROC and PR analyses were described in Supplementary Materials 7). The radiomic and integrated model showed good calibration in the validation cohort (Figure 3(e)). The accuracy, AUROC, and AUPR of Rad-prob were 83%, 0.856, and 0.381. Inte-prob achieved improved performance with an accuracy of 93%, an AUROC of 0.883, and an AUPR of 0.494 (Table 3 and Figures 3(f) and 3(g)). The discrimination of radiomic and integrated models was significantly better than that of the clinical model (*p*=0.036 and 0.013). DCA showed that Inte-prob had the highest clinical value, followed by Rad-prob. Both Rad-prob and Inte-prob were significantly positively correlated with the number of involved LNs in the entire cohort (*r* = 0.442 and 0.464, both *p* < 0.001) (Supplementary Materials 8).

### 3.5. Model Validation in PTMC and CPTC

**Figure 4:**
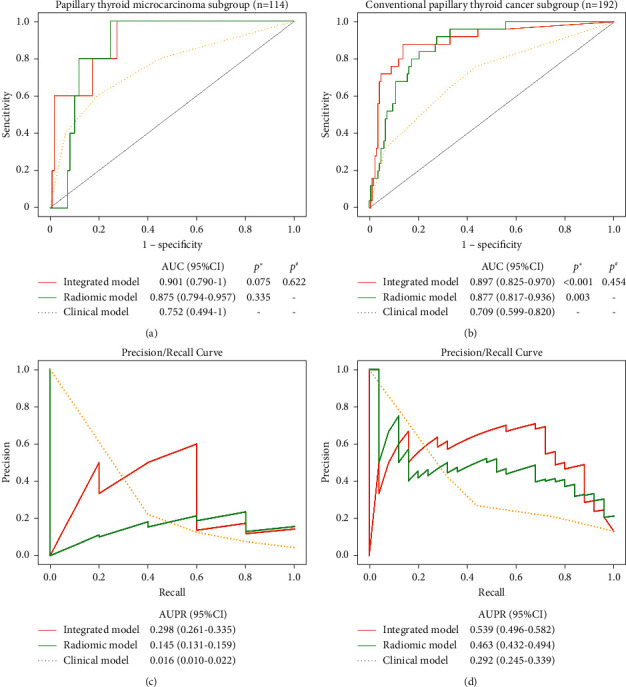
ROC curves and PR curves of the radiomic model and integrated model in the PTMC subgroup (a, c) and CPTC subgroup (b, d). ^*∗*^Statistically different (Delong test) from the clinical model. ^#^Statistically different (Delong test) from the radiomic model. AUPR, area under the precision-recall curve; AUROC, area under the receiver operator characteristic curve; CI, confidence interval; CPTC, conventional papillary thyroid cancer; DCA, decision curve analysis; PTMC, papillary thyroid microcarcinoma.

Through further subgroup analysis, the Rad-prob (OR 2.72, 95% CI 1.37–5.40; 2.72, 95% CI 1.88–3.92) and Inte-prob (OR 2.72, 95% CI 1.43–5.18; 2.72, 95% CI 1.93–3.84) were independent predictors in large-volume LNM in the PTMC and CPTC subgroups (all *p* < 0.001). In PTMC subgroup, the predictive performance of Rad-prob (accuracy 87%; AUROC 0.875; AUPR 0.145) and Inte-prob (accuracy 96%; AUROC 0.901; AUPR 0.298) outperformed that of the clinical model (*p*=0.335 and 0.075). In CPTC subgroup, the prediction of Rad-prob (accuracy 83%; AUROC 0.877; AUPR 0.463) and Inte-prob (accuracy 92%; AUROC 0.897; AUPR 0.539) was significantly better than that of the clinical model (*p* < 0.001 and 0.003) (Table 4 and Figure 4).

## 4. Discussion

This study developed and validated the ANN-based radiomic and integrated models to predict large-volume LNM in cN0 PTC patients. Both models showed good discrimination, calibration, and clinical application, which outperformed the clinical model. The integrated model combining ultrasound and clinical information could achieve better outcome predictions than the radiomic model. Furthermore, the radiomic and integrated models had good predictive performance in the PTMC and CPTC subgroups.

Specific characteristics, including the number, size, and extranodal extension of LNs, can stratify the risk of recurrence in PTC. Small-volume subclinical microscopic N1 disease conveys a much smaller risk of recurrence than large-volume clinically apparent macroscopic LNM [29]. The involvement of more than 5 LNs is defined as large-volume LNM, associated with a 19% risk of recurrence and correlated with lung metastasis [5, 6, 30]. Accurately identifying PTC patients with a poor prognosis is essential for selecting appropriate clinical management strategies. However, the sensitivity for preoperatively detecting cervical LNM is deemed low [12, 13]. Thus, it would be helpful to find preoperative predictors beyond ultrasound features to predict the risk of large-volume LNM in cN0 PTC patients.

Age is the most important prognostic factor for thyroid carcinoma [9]. Large-volume LNM is more likely to appear in PTMC patients aged <40 years [10, 11]. Male sex has been identified as a risk factor for thyroid cancer [31]. PTC in men exhibits aggressive behavior and a worse prognosis than PTC in women [32]. Similarly, in our study, young age and male sex were independent risk factors. However, previous studies have not constructed a predictive model based on these clinical factors. In our study, the predictive performance of the clinical model was not ideal.

Radiomics has been recently applied to thyroid nodules, and it performs well in predicting malignancy and LNM [33–36]. Park et al. [36] reported that radiomics could improve the discrimination of thyroid risk classification systems for malignant thyroid nodules and reduce the number of thyroid nodules recommended for biopsy. Li et al. [34] demonstrated that radiomics has a good prediction ability for pathologic LN stages in PTC patients. Jiang et al. [33] found that a shear wave elastography radiomic signature can accurately predict LNM in PTC patients. Therefore, radiomics is a potential tool to predict large-volume LNM.

Moreover, we used a combination of radiomics and ANN to improve the performance of predictive models. The ANN has several characteristics, including nonlinear statistics, a highly interconnected set of processing units (neurons), and weighted connections [37]. As a commonly used machine learning method, ANN has become a potential tool for predicting clinical outcomes. Hanai et al. [38] demonstrated that ANN is a more helpful tool than conventional statistical methods for predicting the survival of patients with non-small-cell lung cancer. Tong et al. [39] found that ANN-based models showed better performance than logistic regression models in predicting the survival of unresectable pancreatic cancer patients. Our study developed an ANN consisting of an input layer, a hidden layer, and an output layer for large-volume LNM prediction models by inputting the most valuable radiomic features. Although the comparison is not statistically significant due to data imbalance and relatively small study population, from the ROC and PR curves analyses, the ANN-based radiomic model had better discrimination and fewer overfitting possibilities than linear discriminant analysis, support vector machine, and random forest classifiers. The radiomic model showed favorable calibration and predictive value in predicting large-volume LNM in cN0 PTC patients. The novel model improved the clinical model AUROC, AUPR, accuracy, sensitivity, specificity, PPV, and NPV.

Furthermore, the integrated model obtained higher predictive performance by adding clinical independent risk factors. The integrated model displayed good calibration and discrimination with the highest AUROC, AUPR, accuracy, specialty, and PPV among the 3 models. It is worth noting that the PPV value of integrated model was much higher than that of the radiomic model and clinical model. DCA showed that the integrated model gained the highest overall net benefit, followed by the radiomic model. The better performance of radiomic and integrated models in this study indicates that ANN-based models could accurately identify high-risk patients with large-volume LNM, thus providing information to guide treatment and the prognosis for cN0 PTC.

Rad-prob and Inte-prob, unlike the clinical factors, were stable and independent predictors in the PTMC and CPTC subgroups. Young age (OR 3.44, 95% CI 1.40–8.43; *p*=0.007) and male sex (OR 3.26, 95% CI 1.31–8.08; *p*=0.011) were independent risk factors in the CPTC subgroup; however, tumor size (OR 2.07, 95% CI 1.07–3.99; *p*=0.031) was an independent risk factor in the PTMC subgroup. The radiomic and integrated models had stronger predictive value than the clinical model in both subgroups, although the difference was not statistically significant in the PTMC subgroup because of the small subgroup population. Our studies have demonstrated that the radiomic and integrated model could predict large-volume LNM in PTC with different tumor sizes.

Our study has several limitations. First, this study is a single-center retrospective study; thus, selection bias may be inevitable. A prospective multicenter study is necessary to validate the models further. Second, the proportion of large-volume LNM was low, which led to imbalanced data in our study. Although SMOTE was used, this data imbalance inevitably impacts the model construction. Third, the prognosis prediction of the models should be further validated by follow-up of recurrences in the future. Fourth, images were only acquired with Philips ultrasound instruments. We should investigate the influence of images from different ultrasound instruments.

## 5. Conclusions

In conclusion, radiomics can improve the performance of independent clinical predictors in outcome prediction. The ANN-based ultrasound radiomic model and integrated model combining imaging and clinical information have the potential to predict large-volume LNM in cN0 PTC patients preoperatively.

## Figures and Tables

**Table 1:** Baseline clinical and pathological characteristics of the study cohort.

Characteristic	Training cohort (*n* = 183)	Validation cohort (*n* = 123)	*p* value
Clinical characteristics			
Age (years)			
<40	65 (35.5)	43 (35.0)	1.000
≥40	118 (64.5)	80 (65.0)	
Gender			
Male	41 (22.4)	24 (19.5)	0.643
Female	142 (77.6)	99 (80.5)	
Capsule invasion			
Positive	71 (38.8)	39 (31.7)	0.252
Negative	112 (61.2)	84 (68.3)	
Tumor size (mm)	12 (7–14)	12 (9–14)	0.967
ACR TI-RADS score	9 (7–10)	9 (7–10)	0.705
ACR TI-RADS grade			
TR 1–4	30 (16.4)	23 (18.7)	0.713
TR 5	153 (83.6)	100 (81.3)	
Pathological characteristics			
Pathologic *T* stage			
pT1	157 (85.8)	99 (80.5)	0.197
pT2	17 (9.3)	11 (8.9)	
pT3	7 (3.8)	12 (9.8)	
pT4	2 (1.1)	1 (0.8)	
Pathologic N stage			
pN0	118 (64.5)	83 (67.5)	0.786
pN1a	60 (32.8)	38 (30.9)	
pN1b	5 (2.7)	2 (1.6)	
Number of LNs removed ^*∗*^			
Central	9 (6–14)	8 (6–13)	0.972
Lateral^†^	12 (3–33)	32 (22–34)	0.352
Number of positive LNs			
Central	3 (1–6)	3 (2–7)	0.986
Lateral^†^	4 (2–5)	2 (2–5)	0.571
Large-volume LNM			
Positive	19 (10.4)	11 (8.9)	0.827
Negative	164 (89.6)	112 (91.1)	
Multifocality			
Positive	91 (49.7)	55 (44.7)	0.457
Negative	92 (50.3)	68 (55.3)	
Hashimoto's thyroiditis			
Positive	67 (36.6)	41 (33.3)	0.641
Negative	116 (63.4)	82 (66.7)	

^*∗*^6 and 4 in the training and validation cohorts received additional lateral LN dissection; ^†^bilateral. ACR TI-RADS, American College of Radiology Thyroid Imaging, Reporting and Data System; LNM, lymph node metastasis.

**Table 2:** Baseline clinical and pathological characteristics of patients by large-volume LNM status.

	Training cohort		*p* value	Validation cohort		*p* value
	Positive (*n* = 19)	Negative (*n* = 164)		Positive (*n* = 11)	Negative (*n* = 112)	
Clinical characteristics						
Age (years)						
<40	12 (63.2)	53 (32.3)	0.016	7 (63.6)	36 (32.1)	0.049
≥40	7 (36.8)	111 (67.7)		4 (36.4)	76 (67.9)	
Gender						
Male	9 (47.4)	32 (19.5)	0.014	5 (45.5)	19 (17.0)	0.038
Female	10 (52.6)	132 (80.5)		6 (54.5)	93 (83.0)	
Capsule invasion						
Positive	14 (73.7)	98 (59.8)	0.352	6 (54.5)	78 (69.6)	0.492
Negative	5 (26.3)	66 (40.2)		5 (45.5)	34 (30.4)	
Tumor size (mm)	12 (7–14)	12 (7–14)	0.215	12 (12–15)	12 (9–14)	0.357
ACR TI-RADS score	9 (8–10)	9 (7–10)	0.351	9 (7–9)	9 (7–10)	0.699
ACR TI-RADS grade						
TR 1–4	0 (0)	30 (18.3)	0.047	1 (9.1)	22 (19.6)	0.688
TR 5	19 (100)	134 (81.7)		10 (90.9)	90 (80.4)	
Pathological characteristics						
Pathologic T stage						
pT1	15 (78.9)	142 (86.6)	0.528	9 (81.8)	90 (80.4)	1.000
pT2	3 (15.8)	14 (8.5)		1 (9.1)	10 (8.9)	
pT3	1 (5.3)	6 (3.7)		1 (9.1)	11 (9.8)	
pT4	0 (0)	2 (1.2)		0 (0)	1 (0.9)	
Pathologic N stage						
pN0	0 (0)	118 (72.0)	<0.001	0 (0)	83 (74.1)	<0.001
pN1a	19 (100)	41 (25.0)		9 (91.8)	29 (25.9)	
pN1b	0 (0)	5 (3.0)		2 (18.2)	0 (0)	
Multifocality						
Positive	10 (52.6)	81 (49.4)	0.980	6 (54.5)	49 (43.8)	0.712
Negative	9 (47.4)	83 (50.6)		5 (45.5)	63 (56.2)	
Hashimoto's thyroiditis						
Positive	7 (36.8)	60 (36.6)	1.000	6 (54.5)	35 (31.2)	0.219
Negative	12 (63.2)	104 (63.4)		5 (45.5)	77 (68.8)	

ACR TI-RADS, American College of Radiology Thyroid Imaging, Reporting and Data System.

**Table 3:** Performance of the radiomic model and integrated model for predicting large-volume LNM in the training and validation cohorts.

	AUROC (95% CI)	AUPR (95% CI)	ACC (95% CI)	SEN (95% CI)	SPE (95% CI)	PPV (95% CI)	NPV (95% CI)
Integrated model	0.910 (0.853–0.967)^*∗*^	0.463 (0.434–0.492)	93 (88–96)	68 (43–87)	96 (91–98)	65 (41–85)	96 (92–99)
Radiomic model	0.890 (0.837–0.942)^*∗*^	0.348 (0.326–0.370)	86 (80–91)	74 (49–91)	87 (81–92)	40 (24–58)	97 (92–99)
Clinical model	0.714 (0.590–0.838)	0.255 (0.210–0.300)	77 (70–83)	47 (24–71)	80 (74–86)	22 (11–38)	93 (87–97)
Integrated model	0.883 (0.744–1)^*∗*^	0.494 (0.410–0.578)	93 (88–97)	64 (31–89)	96 (91–99)	64 (31–89)	96 (91–99)
Radiomic model	0.856 (0.753–0.958)^*∗*^	0.381 (0.333–0.428)	83 (75–89)	73 (39–94)	84 (76–90)	31 (14–52)	97 (91–99)
Clinical model	0.702 (0.529–0.876)	0.226 (0.171–0.281)	80 (71–86)	45 (17–77)	83 (75–89)	21 (7–42)	94 (87–98)

^*∗*^Significantly different (Delong test) *p* < 0.05 from the clinical model. AUPR, area under the precision-recall curve; AUROC, area under the receiver operator characteristic curve; ACC, accuracy; CI, confidence interval; NPV, negative predictive value; PPV, positive predictive value; SEN, sensitivity; SPE, specificity.

**Table 4:** Performance of the radiomic model and integrated model for predicting large-volume LNM in the PTMC and CPTC subgroups.

	AUROC (95% CI)	AUPR (95% CI)	ACC (95% CI)	SEN (95% CI)	SPE (95% CI)	PPV (95% CI)	NPV (95% CI)
PTMC subgroup (*n* = 114)							
Integrated model	0.901 (0.790–1)	0.298 (0.261–0.335)	96 (90–99)	60 (15–95)	97 (92–99)	50 (12–88)	98 (93–100)
Radiomic model	0.875 (0.794–0.957)	0.145 (0.131–0.159)	87 (79–93)	67 (22–96)	88 (80–93)	24 (7–50)	98 (93–100)
Clinical model	0.752 (0.494–1)	0.016 (0.010–0.022)	80 (71–87)	60 (15–95)	81 (72–88)	12 (3–32)	98 (92–100)
CPTC subgroup (*n* = 192)							
Integrated model	0.897 (0.825–0.970)^*∗*^	0.539 (0.496–0.582)	92 (87–95)	68 (46–85)	95 (91–98)	68 (46–85)	95 (91–98)
Radiomic model	0.877 (0.817–0.936)^*∗*^	0.463 (0.432–0.494)	83 (77–88)	72 (51–88)	84 (78–90)	41 (26–57)	95 (90–98)
Clinical model	0.704 (0.593–0.814)	0.292 (0.245–0.339)	77 (70–83)	44 (24–65)	82 (75–88)	27 (14–43)	91 (85–95)

^*∗*^Significantly different (Delong test) *p* < 0.05 from clinical model. AUPR, area under the precision-recall curve; AUROC, area under the receiver operator characteristic curve; ACC, accuracy; CI, confidence interval; CPTC, conventional papillary thyroid cancer; NPV, negative predictive value; PPV, positive predictive value; PTMC, papillary thyroid microcarcinoma; SEN, sensitivity; SPE, specificity.

## Data Availability

The data used to support the findings of this study are available from the corresponding author upon request.

## Abbreviations

ANN: Artificial neural network
ACR TI-RADS: American College of Radiology Thyroid Imaging, Reporting and Data System
AUPR: Area under the precision-recall curve
AUROC: Area under the receiver operator characteristic curve
CI: Confidence interval
cN0: Clinically N-negative
cN1: Clinically LN-positive
CPTC: Conventional papillary thyroid cancer
DCA: Decision curve analysis
DICOM: Digital Imaging and Communications in Medicine
ICC: Intraclass correlation coefficient
IQR: Interquartile range
LASSO: Least absolute shrinkage and selection operator
LNM: Lymph node metastasis
NPV: Negative predictive value
OR: Odds ratio
PACS: Picture Archiving and Communication Systems
PPV: Positive predictive value
PTC: Papillary thyroid carcinoma
PTMC: Papillary thyroid microcarcinoma
ROI: Region of interest
WHO: World Health Organization.

## Supplementary Materials

Supplementary Materials Supplementary Materials 1: graphs show manually segmented the target nodule region of interest. Supplementary Materials 2: the details of radiomic features extracted. Supplementary Materials 3: the details of the artificial neural network. Supplementary Materials 4: intraobserver and interobserver agreement based on the interclass correlation coefficient of radiomic features. Supplementary Materials 5: least absolute shrinkage and selection operator coefficient profiles of radiomic features. Supplementary Materials 6: training and validation accuracy and loss of the radiomic model and integrated model. Supplementary Materials 7: performance of the ANN-based and conventional machine learning-based classifiers for predicting large-volume LNM according to the ROC and PR analyses. Supplementary Materials 8: correlation of probabilities predicted by the radiomic model and integrated model with a number of positive lymph nodes.

## References

[1] Bray F., Ferlay J., Soerjomataram I., Siegel R. L., Torre L. A., Jemal A. (2018). Global cancer statistics 2018: GLOBOCAN estimates of incidence and mortality worldwide for 36 cancers in 185 countries. *CA: A Cancer Journal for Clinicians*.

[2] Thompson L. (2006). World Health Organization classification of tumours: pathology and genetics of head and neck tumours. *Ear, Nose and Throat Journal*.

[3] Scheumann G. F. W., Gimm O., Wegener G., Hundeshagen H., Dralle H. (1994). Prognostic significance and surgical management of locoregional lymph node metastases in papillary thyroid cancer. *World Journal of Surgery*.

[4] Wada N., Duh Q.-Y., Sugino K. (2003). Lymph node metastasis from 259 papillary thyroid microcarcinomas. *Annals of Surgery*.

[5] Leboulleux S., Rubino C., Baudin E. (2005). Prognostic factors for persistent or recurrent disease of papillary thyroid carcinoma with neck lymph node metastases and/or tumor extension beyond the thyroid capsule at initial diagnosis. *Journal of Clinical Endocrinology and Metabolism*.

[6] Sugitani I., Kasai N., Fujimoto Y., Yanagisawa A. (2004). A novel classification system for patients with PTC: addition of the new variables of large 3 cm or greater nodal metastases and reclassification during the follow-up period. *Surgery*.

[7] Bonnet S., Hartl D., Leboulleux S. (2009). Prophylactic lymph node dissection for papillary thyroid cancer less than 2 cm: implications for radioiodine treatment. *Journal of Clinical Endocrinology and Metabolism*.

[8] Hughes D. T., White M. L., Miller B. S., Gauger P. G., Burney R. E., Doherty G. M. (2010). Influence of prophylactic central lymph node dissection on postoperative thyroglobulin levels and radioiodine treatment in papillary thyroid cancer. *Surgery*.

[9] Huang J., Song M., Shi H. (2021). uPredictive factor of large-volume central lymph node metastasis in clinical n0 papillary thyroid carcinoma patients underwent total thyroidectomy. *Frontiers in Oncology*.

[10] Liu C., Liu Y., Zhang L. (2019). Risk factors for high-volume lymph node metastases in cN0 papillary thyroid microcarcinoma. *Gland Surgery*.

[11] Oh H.-S., Park S., Kim M. (2017). Young age and male sex are predictors of large-volume central neck lymph node metastasis in clinical N0 papillary thyroid microcarcinomas. *Thyroid*.

[12] Roh J.-L., Park J.-Y., Kim J.-M., Song C.-J. (2009). Use of preoperative ultrasonography as guidance for neck dissection in patients with papillary thyroid carcinoma. *Journal of Surgical Oncology*.

[13] Solorzano C. C., Carneiro D. M., Ramirez M., Lee T. M., Irvin G. L. (2004). Surgeon-performed ultrasound in the management of thyroid malignancy. *The American Surgeon*.

[14] Gillies R. J., Kinahan P. E., Hricak H. (2016). Radiomics: images are more than pictures, they are data. *Radiology*.

[15] Cao Y., Zhong X., Diao W., Mu J., Cheng Y., Jia Z. (2021). Radiomics in differentiated thyroid cancer and nodules: explorations, application, and limitations. *Cancers*.

[16] Chen L.-D., Li W., Xian M.-F. (2020). Preoperative prediction of tumour deposits in rectal cancer by an artificial neural network-based US radiomics model. *European Radiology*.

[17] Choi J. S., Kim J., Kwak J. Y., Kim M. J., Chang H. S., Kim E.-K. (2009). Preoperative staging of papillary thyroid carcinoma: comparison of ultrasound imaging and CT. *American Journal of Roentgenology*.

[18] Kim E., Park J. S., Son K.-R., Kim J.-H., Jeon S. J., Na D. G. (2008). Preoperative diagnosis of cervical metastatic lymph nodes in papillary thyroid carcinoma: comparison of ultrasound, computed tomography, and combined ultrasound with computed tomography. *Thyroid*.

[19] Lu W., Zhong L., Dong D. (2019). Radiomic analysis for preoperative prediction of cervical lymph node metastasis in patients with papillary thyroid carcinoma. *European Journal of Radiology*.

[20] Huang X., Wu Z., Zhou A. (2021). Nomogram combining radiomics with the American college of radiology thyroid imaging reporting and data system can improve predictive performance for malignant thyroid nodules. *Frontiers in Oncology*.

[21] Tessler F. N., Middleton W. D., Grant E. G. (2017). ACR thyroid imaging, reporting and data system (TI-RADS): white paper of the ACR TI-RADS committee. *Journal of the American College of Radiology*.

[22] Fedorov A., Beichel R., Kalpathy-Cramer J. (2012). 3D slicer as an image computing platform for the quantitative imaging network. *Magnetic Resonance Imaging*.

[23] van Griethuysen J. J. M., Fedorov A., Parmar C. (2017). Computational radiomics system to decode the radiographic phenotype. *Cancer Research*.

[24] Chawla N. V., Bowyer K. W., Hall L. O., Kegelmeyer W. P. (2002). SMOTE: synthetic minority over-sampling technique. *Journal of Artificial Intelligence Research*.

[25] Huang Y.-q., Liang C.-h., He L. (2016). Development and validation of a radiomics nomogram for preoperative prediction of lymph node metastasis in colorectal cancer. *Journal of Clinical Oncology*.

[26] Li Y., Hong H. G., Ahmed S. E., Li Y. (2019). Weak signals in high-dimensional regression: detection, estimation and prediction. *Applied Stochastic Models in Business and Industry*.

[27] Li W., Guo Q. (2021). Plotting receiver operating characteristic and precision-recall curves from presence and background data. *Ecology and Evolution*.

[28] Kramer A. A., Zimmerman J. E. (2007). Assessing the calibration of mortality benchmarks in critical care: the hosmer-lemeshow test revisited. *Critical Care Medicine*.

[29] Randolph G. W., Duh Q.-Y., Heller K. S. (2012). The prognostic significance of nodal metastases from papillary thyroid carcinoma can be stratified based on the size and number of metastatic lymph nodes, as well as the presence of extranodal extension. *Thyroid*.

[30] Machens A., Dralle H. (2012). Correlation between the number of lymph node metastases and lung metastasis in papillary thyroid cancer. *Journal of Clinical Endocrinology and Metabolism*.

[31] Lee Y. H., Lee Y. M., Sung T. Y. (2017). Is male gender a prognostic factor for papillary thyroid microcarcinoma?. *Annals of Surgical Oncology*.

[32] Ding J., Wu W., Fang J., Zhao J., Jiang L. (2020). Male sex is associated with aggressive behaviour and poor prognosis in Chinese papillary thyroid carcinoma. *Scientific Reports*.

[33] Jiang M., Li C., Tang S. (2020). Nomogram based on shear-wave elastography radiomics can improve preoperative cervical lymph node staging for papillary thyroid carcinoma. *Thyroid*.

[34] Li F., Pan D., He Y. (2020). Using ultrasound features and radiomics analysis to predict lymph node metastasis in patients with thyroid cancer. *BMC Surgery*.

[35] Liang J., Huang X., Hu H. (2018). Predicting malignancy in thyroid nodules: radiomics score versus 2017 American college of radiology thyroid imaging, reporting and data system. *Thyroid*.

[36] Park V. Y., Lee E., Lee H. S. (2021). Combining radiomics with ultrasound-based risk stratification systems for thyroid nodules: an approach for improving performance. *European Radiology*.

[37] Naguib R., Robinson M., Neal D., Hamdy F. (1998). Neural network analysis of combined conventional and experimental prognostic markers in prostate cancer: a pilot study. *British Journal of Cancer*.

[38] Hanai T., Yatabe Y., Nakayama Y. (2003). Prognostic models in patients with non-small-cell lung cancer using artificial neural networks in comparison with logistic regression. *Cancer Science*.

[39] Tong Z., Liu Y., Ma H. (2020). Development, validation and comparison of artificial neural network models and logistic regression models predicting survival of unresectable pancreatic cancer. *Frontiers in Bioengineering and Biotechnology*.

