# Supplementary material for: Artificial Neural Network-Based Ultrasound Radiomics Can Predict Large-Volume Lymph Node Metastasis in Clinical N0 Papillary Thyroid Carcinoma Patients
Source: J Oncol. 2022 Jun 17;2022:7133972. doi: 10.1155/2022/7133972 (PMC9232339; doi:10.1155/2022/7133972)
Supplement: Supplementary Materials — 1: graphs show manually segmented the target nodule region of interest. Supplementary Materials 2: the details of radiomic features extracted. Supplementary Materials 3: the details of the artificial neural network. Supplementary Materials 4: intraobserver and interobserver agreement based on the interclass correlation coefficient of radiomic features. Supplementary Materials 5: least absolute shrinkage and selection operator coefficient profiles of radiomic features. Supplementary Materials 6: training and validation accuracy and loss of the radiomic model and integrated model. Supplementary Materials 7: performance of the ANN-based and conventional machine learning-based classifiers for predicting large-volume LNM according to the ROC and PR analyses. Supplementary Materials 8: correlation of probabilities predicted by the radiomic model and integrated model with a number of positive lymph nodes. [file 7133972.f1.docx]

**Supplement Material 1**


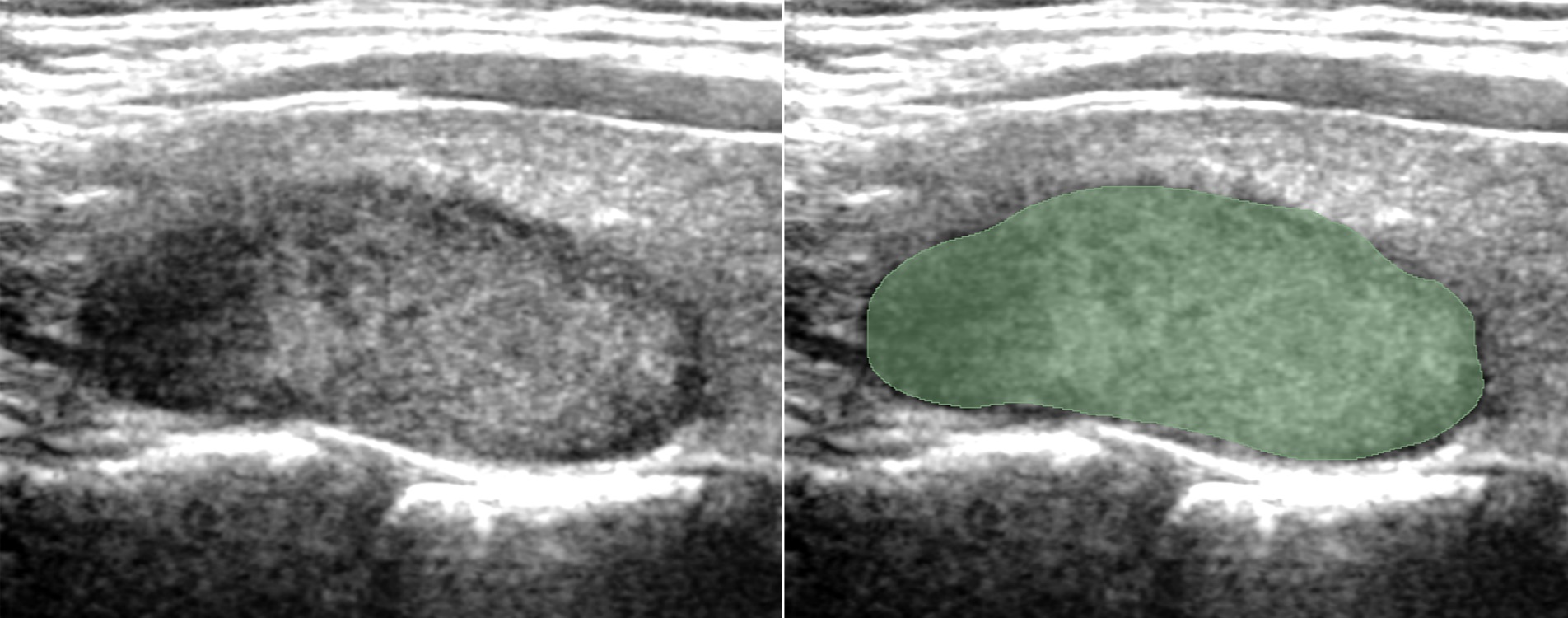


**Figure S1** The grayscale ultrasound image (left) and manually drawn region of interest (right) of papillary thyroid carcinoma (green mask).

**Supplement Material 2** **Radiomic features extracted**

We adopted resampling as a preprocessing method, which was performed to obtain a voxel size of 1 x 1 x 1 mm^3^ via trilinear interpolation before feature calculation. After normalization, 849 radiomic features from original and wavelet-filtered images of each nodule were extracted by Pyradiomics version 3.0.1 (http://www.radiomics.io/pyradiomics.html).

These features could be divided into 3 groups:

1. First-order features (n=18): InterquartileRange, Skewness, Uniformity, Median, Energy, RobustMeanAbsoluteDeviation, MeanAbsoluteDeviation, TotalEnergy, Maximum, RootMeanSquared, 90Percentile, Minimum, Entropy, Range, Variance, 10Percentile, Kurtosis, MeanInterquartileRange, Skewness, Uniformity, Median, Energy, RobustMeanAbsoluteDeviation, MeanAbsoluteDeviation, TotalEnergy, Maximum, RootMeanSquared, 90Percentile, Minimum, Entropy, Range, Variance, 10Percentile, Kurtosis, Mean;
2. Shape features (n=12): VoxelVolume, Maximum3DDiameter, MeshVolume, MajorAxisLength, Sphericity, LeastAxisLength, Elongation, SurfaceVolumeRatio, Maximum2DDiameterSlice, Flatness, SurfaceArea, MinorAxisLength, Maximum2DDiameterColumn, Maximum2DDiameterRow;
3. Textural features (n=75):
4. Gray Level Dependence Matrix (GLDM)- GrayLevelVariance, HighGrayLevelEmphasis, DependenceEntropy, DependenceNonUniformity, GrayLevelNonUniformity, SmallDependenceEmphasis, SmallDependenceHighGrayLevelEmphasis, DependenceNonUniformityNormalized, LargeDependenceEmphasis, LargeDependenceLowGrayLevelEmphasis, DependenceVariance, LargeDependenceHighGrayLevelEmphasis, SmallDependenceLowGrayLevelEmphasis, Low Gray Level Emphasis;
5. Gray Level Co-occurrence Matrix (GLCM)- JointAverage, SumAverage, JointEntropy, ClusterShade, MaximumProbability, Idmn, JointEnergy, Contrast, DifferenceEntropy, InverseVariance, DifferenceVariance, Idn, Idm, Correlation, Autocorrelation, SumEntropy, MCC, SumSquares, ClusterProminence, Imc2, Imc1, DifferenceAverage, Id, ClusterTendency;
6. Gray Level Run Length Matrix (GLRLM)- ShortRunLowGrayLevelEmphasis, GrayLevelVariance, LowGrayLevelRunEmphasis, GrayLevelNonUniformityNormalized, RunVariance, GrayLevelNonUniformity, LongRunEmphasis, ShortRunHighGrayLevelEmphasis, RunLengthNonUniformity, ShortRunEmphasis, LongRunHighGrayLevelEmphasis, RunPercentage, LongRunLowGrayLevelEmphasis, RunEntropy, HighGrayLevelRunEmphasis, RunLengthNonUniformityNormalized;
7. Gray Level Size Zone Matrix (GLSZM)- GrayLevelVariance, ZoneVariance, GrayLevelNonUniformityNormalized, SizeZoneNonUniformityNormalized, SizeZoneNonUniformity, GrayLevelNonUniformity, LargeAreaEmphasis, SmallAreaHighGrayLevelEmphasis, ZonePercentage, LargeAreaLowGrayLevelEmphasis, LargeAreaHighGrayLevelEmphasis, HighGrayLevelZoneEmphasis, SmallAreaEmphasis, LowGrayLevelZoneEmphasis, ZoneEntropy, SmallAreaLowGrayLevelEmphasis
8. Neighbouring Gray Tone Difference Matrix (NGTDM)- Coarseness, Complexity, Strength, Contrast, Busyness.

Wavelet transform was used to analyze the spatial time-frequency of the obtained 2-dimensional images, which were divided into 8 sub-images: HHH, HLL, HLH, HHL, LLH, LHL, LHH, and LLL.

**Supplement Material 3** **Artificial neural network**

An artificial neural network (ANN) is based on a collection of connected units or nodes called artificial neurons, which loosely model the neurons in a biological brain. It is a nonlinear statistical model which can be used to learn representations of the input that capture the salient characteristics of the input distribution. In this study, we conducted all experiments on NVIDIA 2060 RTX, and used Keras (version:2.6.0, https://keras.io/) for building the model. The modeling code was written in Python. We build the model using a single-hidden-layer, feed-forward ANN with a backpropagation algorithm. The adam optimizer with a learning rate weight of 0.001was employed to train the networks. The batch size was set to 32. The epoch was set to 200 for iterations, and Early Stopping was used to avoid overfitting. The number of neurons should be between the input layer size and the output layer size, usually two-thirds of the input size. Thus, the number of neurons in the 1st hidden layer ranges from 12 to 24 to make the models most accurate and generalizable. In our study, the number of neurons was set as 15 and 19 for the radiomic and integrated models, respectively. This study randomly divided the training cohort into train and test groups (6:4) to build models. The validation cohort was used to validate models independently.

**Supplement Material 4**


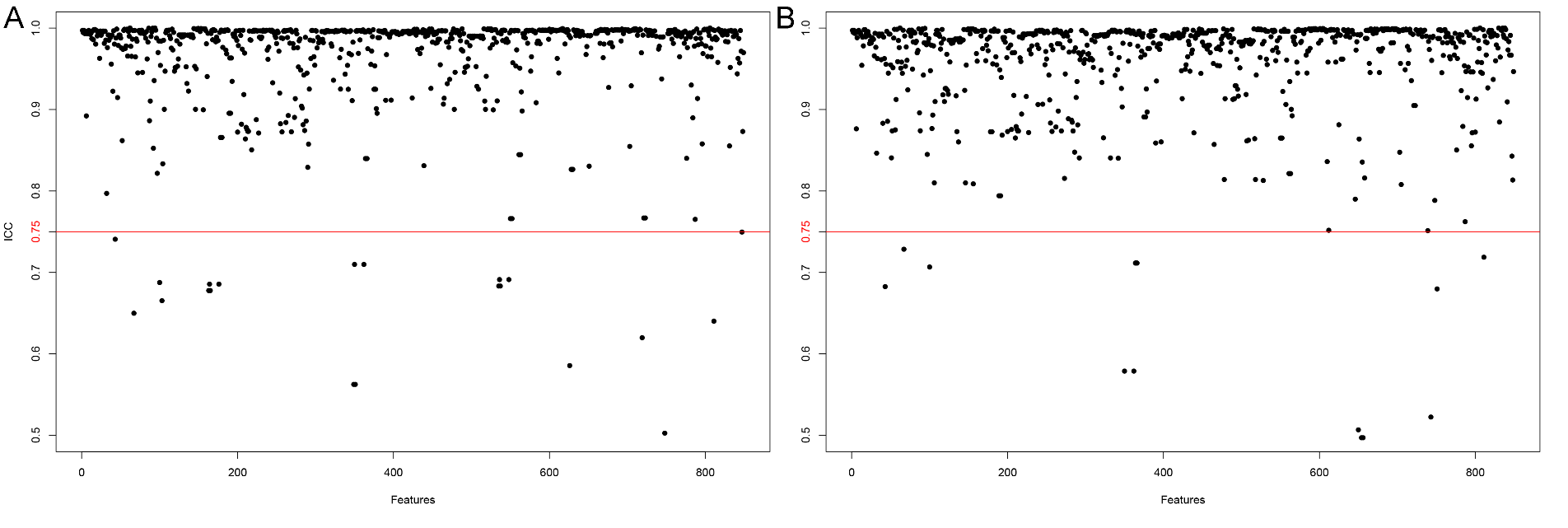


**Figure S2** Intra- and inter-observer agreement based on the interclass correlation coefficient (ICC) of radiomic features. 95.1% (807/849; mean ICC=0.950) and 95.6% (812/849; mean ICC=0.831) radiomic features presented good intra-observer (A) and intra-observer (B) agreement with ICCs of >0.75 (above the red cutoff line).

**Supplement Material 5**


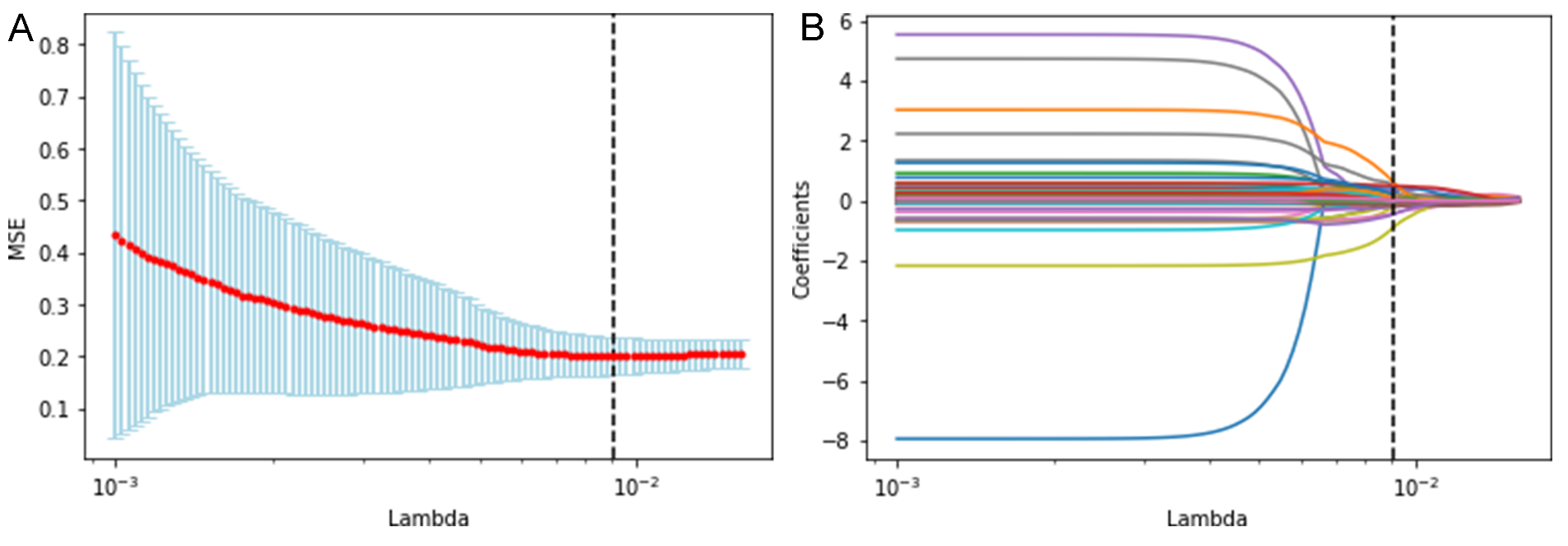


**Figure S3** Least absolute shrinkage and selection operator (LASSO) coefficient profiles of radiomic features. The LASSO model used the 10-fold cross-validation and the minimal criteria to generate the optimal penalization coefficient lambda (λ). Dotted lines on the left and right denote the minimum criterion. As a result, the λ value of 0.009 was selected.

**Supplement Material 6**


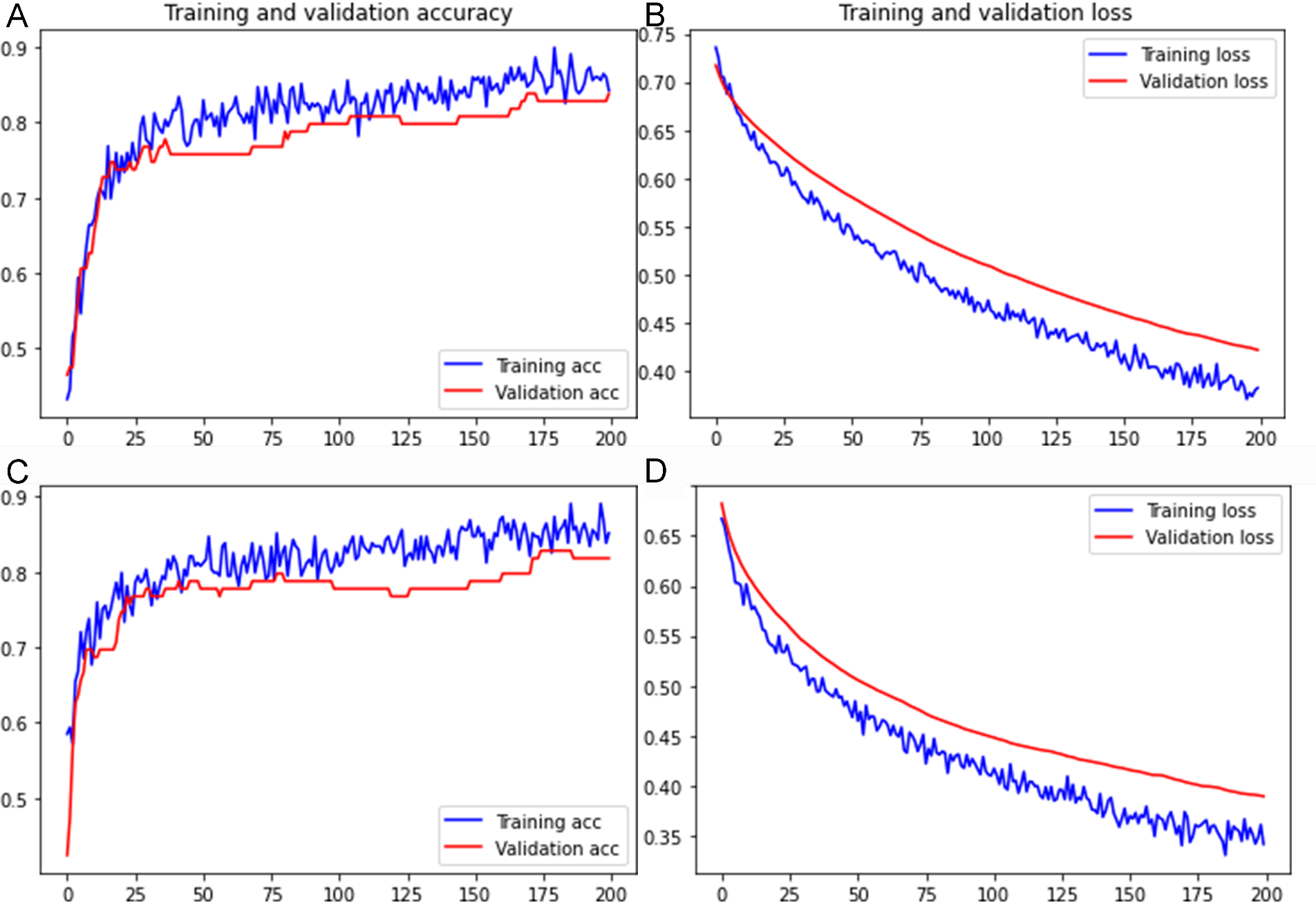


**Figure S4** Training and validation accuracy and loss in large-volume

lymph node metastasis (LNM) classification based on ANN radiomic model (A, B) and integrated model (C, D).

**Supplement Material 7**

Table 1: Performance of the ANN-based and conventional machine learning-based classifiers for predicting large-volume LNM.

|  | AUROC (95% CI) | | AUPR (95% CI) | |
| --- | --- | --- | --- | --- |
|  | Training cohort | Validation cohort | Training cohort | Validation cohort |
| LDA | 0.865(0.793-0.937) | 0.806(0.664-0.948) | 0.344(0.315-0.373) | 0.272(0.225-0.319) |
| RF | 0.985(0.960-1) | 0.759(0.616-0.902) | 0.868(0.846-0.890) | 0.206(0.167-0.245) |
| SVM-RBF | 0.999(0.998-1) | 0.775(0.676-0.874) | 0.968(0.966-0.970) | 0.182(0.158-0.206) |
| ANN | 0.890(0.837-0.942) | 0.856(0.753-0.958) | 0.348(0.326-0.370) | 0.381(0.333-0.428) |

Abbreviations: ANN, artificial neural network; AUPR, area under the precision-recall curve; AUROC, area under the receiver operator characteristic curve; CI, confidence interval; LDA, linear discriminant analysis; LNM, lymph node metastasis; SVC-RBF, support vector machine with radial basis function; RF, random forest.

**
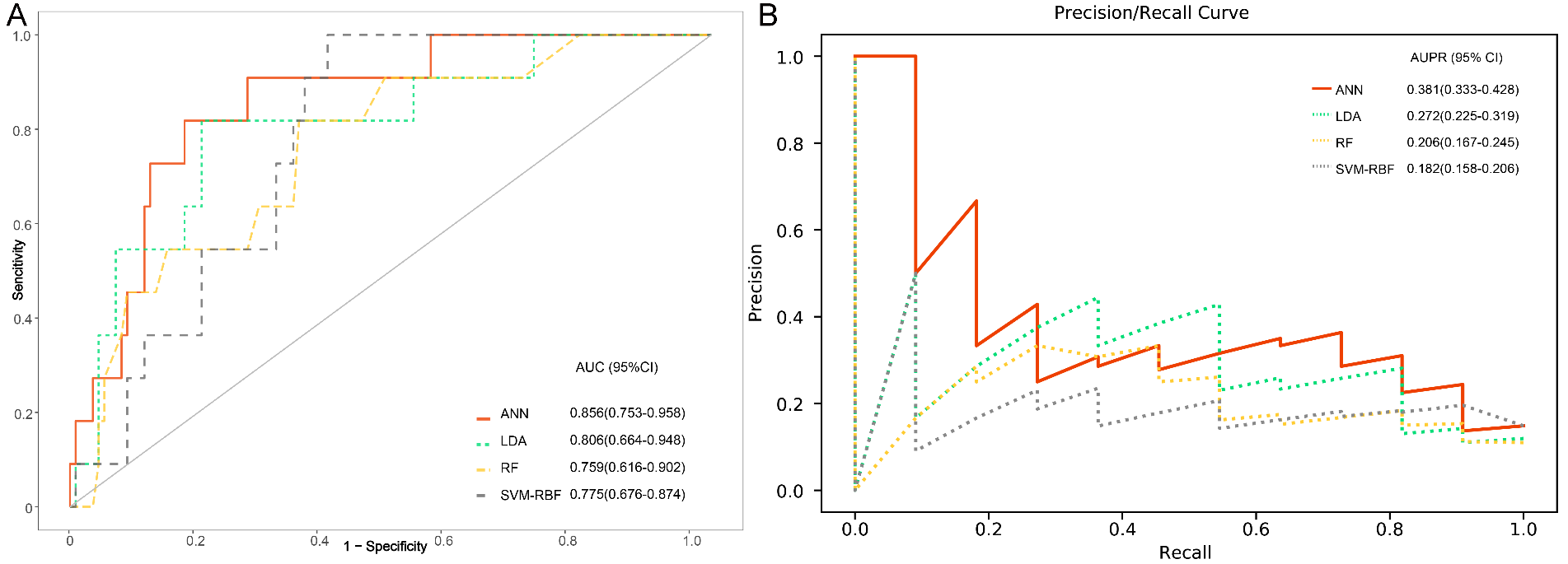
**

**Figure S6** ROC curves (A) and PR curves (B) of ANN-based and conventional machine learning-based classifiers for predicting large-volume LNM in the validation cohort.

Abbreviations: ANN, artificial neural network; AUPR, area under the precision-recall curve; AUROC, area under the receiver operator characteristic curve; CI, confidence interval; LDA, linear discriminant analysis; LNM, lymph node metastasis; SVC-RBF, support vector machine with radial basis function; RF, random forest.

**Supplement Material 8**


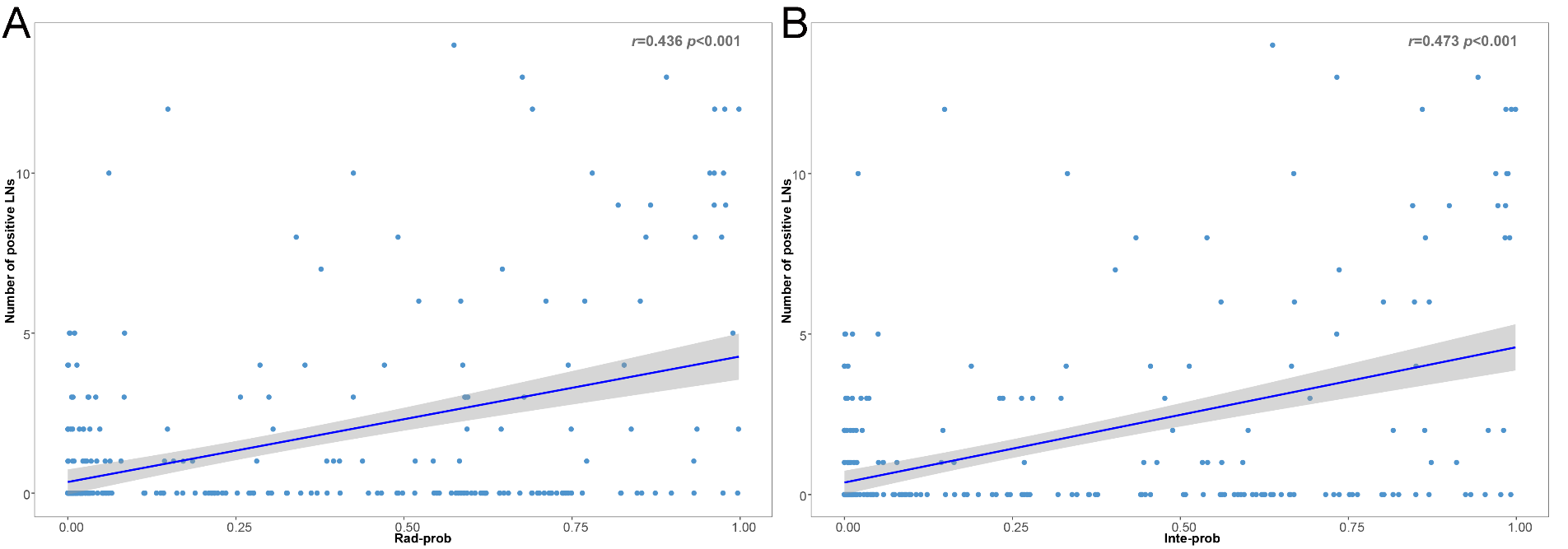


**Figure S7** Correlation of Rad-prob (A) and Inte-prob (B) with number of positive lymph nodes.
